# Transcriptomic profile of adverse neurodevelopmental outcomes after neonatal encephalopathy

**DOI:** 10.1038/s41598-020-70131-w

**Published:** 2020-08-04

**Authors:** Paolo Montaldo, Aubrey Cunnington, Vania Oliveira, Ravi Swamy, Prathik Bandya, Stuti Pant, Peter J. Lally, Phoebe Ivain, Josephine Mendoza, Gaurav Atreja, Vadakepat Padmesh, Mythili Baburaj, Monica Sebastian, Indiramma Yasashwi, Chinnathambi Kamalarathnam, Rema Chandramohan, Sundaram Mangalabharathi, Kumutha Kumaraswami, Shobha Kumar, Naveen Benakappa, Swati Manerkar, Jayashree Mondhkar, Vinayagam Prakash, Mohammed Sajjid, Arasar Seeralar, Ismat Jahan, Sadeka Choudhury Moni, Mohammod Shahidullah, Radhika Sujatha, Manigandan Chandrasekaran, Siddarth Ramji, Seetha Shankaran, Myrsini Kaforou, Jethro Herberg, Sudhin Thayyil

**Affiliations:** 1 Department of Brain Sciences, Centre for Perinatal Neuroscience, Imperial College London, London, UK; 2 Neonatal Unit, Università Degli Studi Della Campania “Luigi Vanvitelli”, Naples, Italy; 3 Paediatric Infectious Diseases, Department of Infectious Diseases, Imperial College London, London, UK; 4 Neonatal Medicine, Indira Gandhi Institute of Child Health, Bangalore, Karnataka India; 5 Neonatal Medicine, Institute of Obstetrics and Gynaecology, Madras Medical College, Chennai, Tamil Nadu India; 6 Neonatal Medicine, Institute of Child Health, Madras Medical College, Tamil Nadu, Chennai, India; 7 Neonatal Medicine, Sion Medical College, Mumbai, India; 8 Neonatal Medicine, Bangabandhu Sheikh Mujib Medical University, Dhaka, Bangladesh; 9 Neonatal Medicine, Government Medical College, Thiruvananthapuram, Kerala India; 10 Neonatal Medicine, Maulana Azad Medical College, New Delhi, Delhi India; 11 Neonatal-Perinatal Medicine, Wayne State University, Detroit, MI USA

**Keywords:** Computational biology and bioinformatics, Developmental biology, Molecular biology, Neuroscience

## Abstract

A rapid and early diagnostic test to identify the encephalopathic babies at risk of adverse outcome may accelerate the development of neuroprotectants. We examined if a whole blood transcriptomic signature measured soon after birth, predicts adverse neurodevelopmental outcome eighteen months after neonatal encephalopathy. We performed next generation sequencing on whole blood ribonucleic acid obtained within six hours of birth from the first 47 encephalopathic babies recruited to the Hypothermia for Encephalopathy in Low and middle-income countries (HELIX) trial. Two infants with blood culture positive sepsis were excluded, and the data from remaining 45 were analysed. A total of 855 genes were significantly differentially expressed between the good and adverse outcome groups, of which *RGS1* and *SMC4* were the most significant. Biological pathway analysis adjusted for gender, trial randomisation allocation (cooling therapy versus usual care) and estimated blood leukocyte proportions revealed over-representation of genes from pathways related to melatonin and polo-like kinase in babies with adverse outcome. These preliminary data suggest that transcriptomic profiling may be a promising tool for rapid risk stratification in neonatal encephalopathy. It may provide insights into biological mechanisms and identify novel therapeutic targets for neuroprotection.

## Introduction

Neonatal encephalopathy (NE) related to perinatal asphyxia is the most common cause of death and neurodisability in term babies with an incidence of 2 to 3 per 1,000 live births in high-income countries, and 10 to 20 per 1,000 livebirths in low and middle-income countries^1,2^. In high income countries, therapeutic hypothermia partially improves outcomes, although adverse outcomes still occur in up to 30% of the cooled infants^3^.

As the underlying brain injury evolves over hours and days after birth, early identification of at-risk encephalopathic infants is challenging and often inaccurate, particularly in cooled infants^4^. Furthermore, NE is heterogenous condition resulting from a multitude of aetiologies including acute or sub-acute perinatal hypoxia, inflammation, infection, metabolic and genetic causes, although these may be clinically indistinguishable^5^. These difficulties have hindered the development of novel neuroprotective strategies. A better understanding of molecular mechanisms of hypoxia-induced brain injury and of the different underlying pathologies has translational potential that may lead to future discoveries and novel neuroprotective therapies.

The use of gene expression for disease stratification and for elucidation of underlying disease mechanisms has previously been shown in sepsis, paediatric tuberculosis and Kawasaki Disease^6–8^. More recently, we have reported that babies with NE have a unique gene expression profile when compared with healthy controls and septic babies, and have an upregulation of the hypoxia inducible transcription factor 1α (HIF1α)^9^. Here, we examined if babies who developed adverse neurodevelopmental outcome after NE, had a unique host blood gene expression profile at birth.

## Results

We analysed the blood samples from the first 47 neonates recruited to a randomised controlled trial of whole body cooling versus usual care—HELIX (Hypothermia for Encephalopathy in Low and Middle-Income Countries) trial^10^. Two neonates with positive blood culture sepsis (*Klebsiella pneumoniae* and *Pseudomonas aeruginosa*) were excluded and therefore 45 infants were included in the analysis. The adverse outcome group was defined as death or moderate or severe disability (motor disability and Bayley III). Neurodevelopmental outcome data, based on Bayley assessments carried-out between 18 to 22 months of age, were available in all infants, of which 23 (51%) had adverse outcome and 22 (49%) had good outcome. No significant demographic differences were found between NE infants with adverse and good outcome (Table 1).

**Table 1:** Baseline clinical characteristics.

Median (IQR) or N (%)	Adverse outcome (n = 23)	Good outcome (n = 22)	P value
Birth weight, g	2,730 (2,487–3,055)	2,932 (2,750–3,225)	0.03
Gestation, weeks	38 (37–40)	39 (38–40)	0.13
Gender (males)	15 (65)	11 (50)	0.35
Apgar 1 min	2 (1–3)	3 (2–3.5)	0.17
Apgar 5 min	5 (4–5)	5 (5–5)	0.10
Encephalopathy grade (moderate/severe)	14/9	20/2	0.01
Death	22 (95)	–	–
Prolonged rupture of membranes (> 24 h)	0	0	–
Maternal pyrexia	1(4)	0	0.1
Invasive Ventilation	20 (87%)	4 (18.2%)	0.0001
Perinatal sentinel events*	0	1(4)	0.3
Persistent pulmonary Hypertension	5 (21.7) (22)	0	0.03 0.02
Hypotension requiring inotropes	23 (100)	13 (59.1%)	0.001
Abnormal clotting	11 (47.8%)	3 (13.6%)	0.02
Anticonvulsants	18 (78.3%)	19 (86.4%)	0.69

*Perinatal sentinel events were defined as one of the following: antepartum haemorrhage, umbilical cord mishaps, shoulder dystocia or ruptured uterus.

Continuous variables were compared by using Mann–Whitney U-test and categorical variables by using Pearson chi-squared test or Fisher exact test.

**Figure 1:**
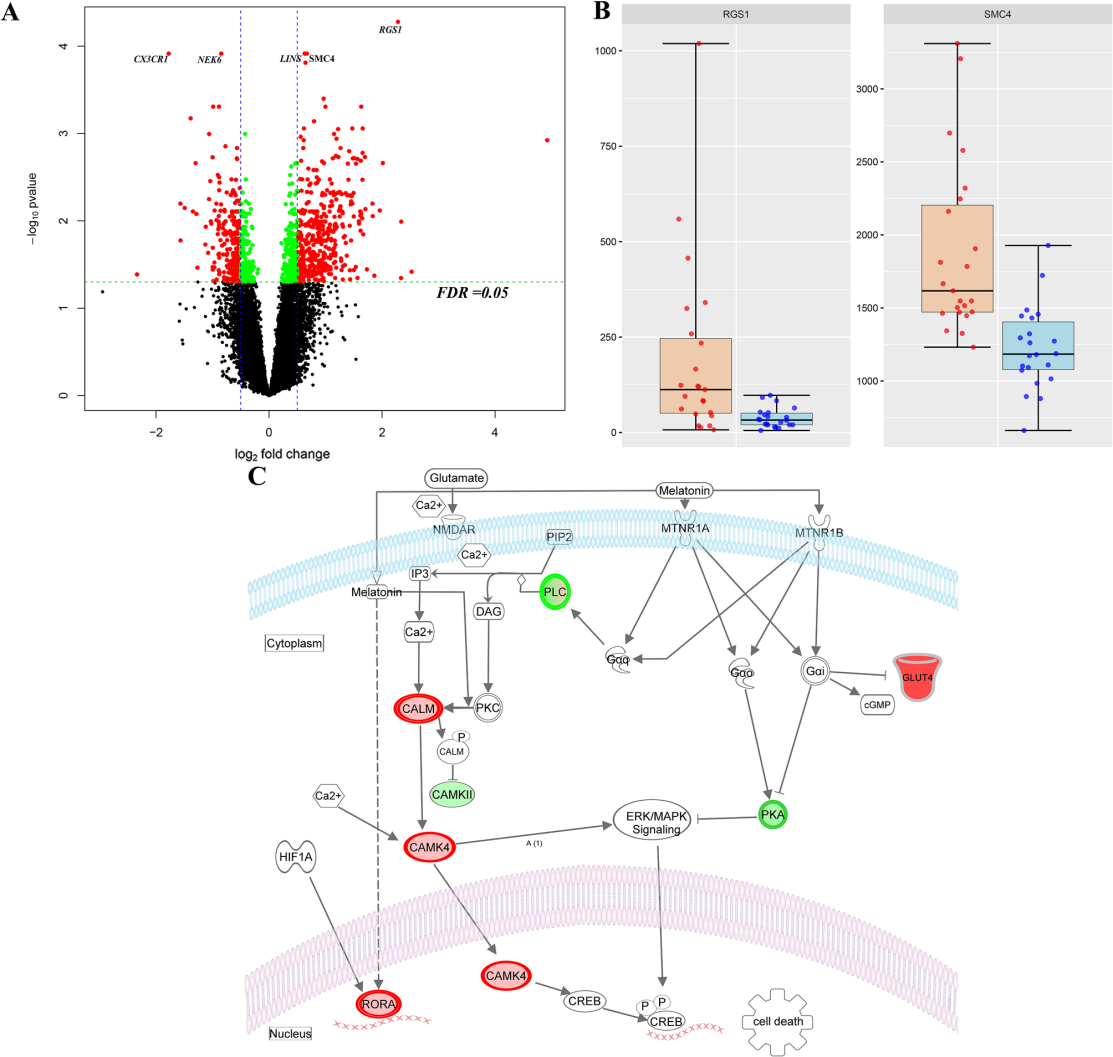
(**A**) Volcano plot showing the significant genes identified in the comparison of neonates with adverse versus good outcome, plotted according to log_2_ fold-change (x axis) and log_10_ p value (y axis). In green are genes with false discovery rate (FDR) < 0.05 and log_2_ fold change < 0.4 in red are genes with FDR < 0.05 and log_2_ fold change > 0.4. (**B**) Box plot (median, IQR) of gene count values expressed as Fragments Per Kilobase of transcript per Million mapped reads (FPKM) (y axis) of the 6 most significant genes for children with normal (blue) compared with abnormal neurodevelopmental outcome at 2 years (orange). (**C**) Brain hypoxia leads to Ca^2+^ influx with activation of the Ca^2+^/calmodulin dependent protein kinase IV (CaMK-IV) cascade. CaMK-IV in the cytosol has a proapoptotic effect and is responsible for hypoxic neural cell death both through activation of MAP kinases signalling in the cytosol and through phosphorylation of cAMP response element-binding protein (CREB) in the nucleus, which enhances the expression of pro-apoptotic proteins. Melatonin binds to its plasma membrane receptor MTNR1, to calmodulin and to nuclear receptor retinoid-related receptor alpha (RORA) increasing its expression. RORA is also considered a downstream target of HIF-1α and its levels have been found upregulated in the cellular response to hypoxia. MTNR1A and MTNR1B activation increases PKC activity through activation of Gαq, which stimulates the PLC signalling cascade and leads to inhibition of Ca^2+^/calmodulin dependent protein kinase (CAMK). Both MTNR1A and MTNR1B activation by melatonin inhibits cAMP formation. Furthermore, activation of MTNR1B decreases the expression of the glucose transporter GLUT4, which in turn decreases glucose uptake. The upregulated genes in our analysis are shown in red, while the downregulated genes are shown in green. *CALM* Calmodulin, *CAMK4* calcium/calmodulin dependent protein kinase 4, *CAMKII* calcium/calmodulin dependent protein kinase II, *CREB* cAMP-response element binding protein, *DAG* diacylglycerol, *ERK* extracellularly regulated kinase, *Gαq* Gq protein alpha subunit, *Gαi* α subunit of the heterotrimeric G protein complex, *GLUT4* Glucose transporter type 4, *Hif-1 alpha* Hypoxia-inducible factor 1-alpha, *PIP2* Phosphatidylinositol biphosphate, *PKA* protein kinase A, *PKC* Protein Kinase C signalling, *MAPK* mitogen-activated protein kinases, *MTNR1A* Melatonin receptor type 1A, *MTNR1B* Melatonin receptor type 1B, *IP3* Inositol trisphosphate, *NMDAR* *N*-methyl-D-aspartate receptor, *RORA* Retinoid-related receptor alpha. **A**,**B** were created using R (version 4.0.0) (https://cran.r-project.org/). **C** Was created through the use of Ingenuity pathway software (QIAGEN Inc., https://www.qiagenbioinformatics.com/products/ingenuity-pathway-analysis).

As the trial is ongoing, authors were masked to the treatment (therapeutic hypothermia) allocation, which was provided as A or B. There was no statistically significant difference in outcome between treatment groups A and B, and the clinical severity of NE in two groups was balanced at the time of randomisation allocation (Supplementary Fig. 1). Differential gene expression analyses comparing infants with adverse and good outcome revealed 855 significant differentially expressed genes with a false discovery rate (FDR) value < 0.05 and an absolute log2 fold change ≥ 0.4, after adjusting for the randomisation allocation and gender (Fig. 1A). Of these genes, 523 (61%) were over-expressed and 332 (38%) under-expressed in NE infants with adverse outcome as shown in the volcano plot.

The most significant gene ordered by FDR was regulator of G-protein signalling 1 (*RGS1*) (FDR value < 0.001, log2 fold change 2.28) followed by structural maintenance of chromosomes protein 4 (*SMC4)* (FDR value < 0.001, log_2_ fold change 0.63) (Fig. 1B).

Finally, we re-analysed the data after adjusting for the randomisation allocation, gender and blood cell type proportions (neutrophils and lymphocyte cell fractions) and identified 660 significant differentially expressed genes with an FDR value < 0.05 and an absolute log2 fold change ≥ 0.4 (Supplementary Fig. 2). Of these genes, 389 (59%) were over-expressed and 271 (41%) under-expressed. The most significant gene remained *RGS1* (FDR value < 0.001, log2 fold change 2.28) together with *SMC4* (FDR value < 0.001, log_2_ fold change 0.63). We then applied pathway enrichment analysis (Ingenuity Pathway Analysis—www.Ingenuity.com) to all the differentially expressed genes (FDR < 0.01 and absolute log_2_ fold change ≥ 0.4). The most statistically significant pathways based on the FDR and ratio values were Mitotic Roles of Polo-Like Kinase (FDR = 0.002, ratio = 0.15) and Melatonin (FDR = 0.003, ratio = 0.11) (Fig. 1C).

We assessed the relationship of genes of interest with different blood cell types (neutrophils and lymphocytes)^11^ and found that *RGS1*, *SMC4,* as well as the differentially expressed genes of the melatonin pathway were most positively correlated with the lymphocytes fraction (Supplementary Table 1).

## Discussion

Our study shows that infants who will go on to develop adverse outcome after NE have a specific gene expression profile, when compared to those who have good outcome. Our data also suggest that polo like kinase and melatonin pathways play a key role in those babies with NE who later develop adverse outcomes. Melatonin is a powerful free radical scavenger, which controls cell death, free radical production, ATP consumption and integrity of the electron transport chain^12,13^. Polo-like kinases instead, are activated by hypoxia/reoxygenation insult and result in a decreasing survival and increasing cell apoptosis^14^.

Despite the widespread use of therapeutic hypothermia as standard treatment in NE, up to 55% of infants with moderate/severe NE are reported to have adverse long-term outcome, in high-income countries^15^. Therefore, there is need to identify adjunct neuroprotective strategies to improve the outcome of infants with moderate or severe NE. Establishing the biological function of differentially expressed genes associated with adverse outcome after neonatal encephalopathy may enable better understanding of brain injury, permitting the identification of new therapeutic targets.

Different pre-clinical studies suggest the potential of melatonin as a neuroprotectant^16^. Melatonin counteracts the effects of hypoxia firstly through the inhibition of ERK/MAK signalling with down regulation of c-AMP response element binding (CREB) protein, secondly through the inhibition Ca^2+^/calmodulin-dependent protein kinases and finally through the upregulation of RORα, which causes a decreased expression of pro-apoptotic proteins and anti-inflammatory effects^17–19^. *RORA* is also considered a HIF-1 target gene and its levels have been found upregulated in the cellular response to hypoxia (Fig. 1C)^20^.

Animal evidence has shown that after hypoxia, there is an increased nuclear Ca^2+^ influx and nuclear Ca^2+^/calmodulin kinase IV (CaMKIV) activity, which enhances the expression of different transcription factors responsible for programmed cell death through the upregulation of CREB protein^21,22^. This latter is responsible for the neuronal apoptosis following cerebral hypoxia and is a crucial point of action of therapeutic hypothermia neuroprotective effects^21–23^. A recent study has underlined the importance of this cascade, showing that its synergic downregulation together with therapeutic hypothermia, potentiates neuroprotection with reduced cell death and improved neuropathology in a piglet hypoxia model^24^. Of note, CaMKIV levels have been found to increase with the increase in the degree of cerebral tissue hypoxia assessed by cerebral tissue high energy phosphates^25^. Therefore, the present upregulation of CaMKIV in our study, may reflect that infants with adverse outcome were those exposed to more severe degrees of cerebral hypoxia.

The polo-like kinase family are cell cycle process regulators, which are important mediators of the cellular responses to hypoxia and reactive oxygen species exposure^26^. Hypoxia-induced HIF-1α expression is strongly associated with a significant down-regulation of polo-like kinases expression in HeLa cells leading to increased cell survival and proliferation^27^. HIF-1 in fact, has as an important role under hypoxic conditions in regulating cell proliferation and metabolism. Taken together, our findings stress the leading role of HIF-1α in NE, which is involved in both the melatonin and polo-like kinase cascades^27–30^ and interacts directly with both RGS1 and SMC4, which are downstream targets of HIF-1α^30,31^. These results are also consistent with our previous data, comparing healthy infants and NE, which showed an upregulation of *HIF1A* and *MALAT1* in NE^9^*. MALAT1* inhibits HIF-1α ubiquitination and in this way, enhances its activity and increases anaerobic glycolysis.

In our study a marked differential expression was found in *RGS1* and *SMC4*. *SMC4* is responsible for regulating chromosome organization and dynamics^32^. Recent studies demonstrated also that *SMC4* is associated with tumor progression and invasion and the expression of *SMC4* is positively correlated with HIF-1a^30^. RGS1 protein is the most abundant RGS protein in the microglia and is a key gene of the immunomodulatory response to neuroinflammation as well as a key target of different immunological and neurodegenerative diseases such as multiple sclerosis, inflammatory bowel and Parkinson’s disease^33,34^. Recent evidence suggests that RGS1 protein is involved in the hypoxia-dependent inflammatory response^35,36^, through activation of the AKT signalling pathway, which is switched-on after chronic moderate hypoxia. A leading role of neuroinflammation is now increasingly recognized in neonatal brain damage following NE^37,38^. Therefore, the high expression levels of this gene around birth in babies with adverse outcome later in life, may reflect the increased hypoxia-dependent neuroinflammation in babies with an adverse outcome.

When we assessed the gene expression signature of different blood cell types, we found that the top differentially expressed genes were expressed the most in lymphocytes. This may reflect the immunomodulatory effect of HIF signal, which has been shown to increase the potency of regulatory T cells by affecting the expression of their transcriptional activator Foxp3^39^.

Our study has several limitations. Firstly, the sample size of the study was small and these findings need to be validated in a larger cohort. Secondly, we examined gene expression at only one timepoint, within six hours after birth. Gene expression profiles are likely to change over time in the post-natal period and therefore our findings may differ if gene expression profiles are examined beyond this time window. Finally, this study was conducted in low-middle income countries and therefore, caution is needed before translating these findings to different settings.

Our research was exploratory without a predefined hypothesis on hypoxic-ischemic injury induced gene expression changes and was intended to examine the early host gene expression profile associated with later adverse outcomes. Our preliminary data suggest that babies with adverse outcome after NE had a characteristic gene expression profile that can be measured in whole blood soon after birth, and that selected transcripts correlated better with final clinical outcome than immediate clinical assessment of NE severity. If these findings can be replicated in larger cohort of babies with NE, it may open new therapeutic avenues and to develop new neuroprotection therapies in the future.

## Materials and methods

### Study design and subjects, samples

The HELIX trial was a multi-country randomised controlled trial of whole-body cooling versus usual care in NE and recruited 408 babies from seven tertiary neonatal units in India, Sri Lanka and Bangladesh between Aug 2016 and Feb 2019^10^. The study was approved by the research ethics committee of Imperial College London, and recruiting hospital sites, and informed parental consent was obtained prior to recruitment. All methods were carried out in accordance with relevant guidelines and regulations.

All babies had a structured neurological examination based on the Eunice Kennedy National Institute of Child health and Human Development (NICHD) system by a certified examiner within six hours of birth and the babies were classified as having moderate or severe encephalopathy^40,41^. We collected 0.5 ml of blood (venous or arterial) from the recruited infants within six hours of birth and prior to initiation of cooling therapy, if randomised to the cooling arm. The blood was gently mixed with 1.4 mL RNA stabilizing solution (PreAnalytiX BD/QIAgen) and subsequently stored in a − 80 °C freezer until analysis. The blood samples from the first 47 infants recruited from three Indian sites were included in this work. We excluded babies with culture positive sepsis from the analysis. A flow-chart of the study is in supplementary Fig. 1.

Between 18 to 22 months of age, a certified examiner performed neurodevelopmental evaluation using Bayley scales of Infant development Version III. The primary outcome was death or moderate or severe disability. We defined severe disability as any of the following: a Bayley III cognitive score < 70; a Gross Motor Function Classification System (GMFCS) level of 3 to 5; blindness; or hearing loss (inability to understand commands despite amplification)^41^. We defined moderate disability as a Bayley III cognitive score of 70 to 84 and either a GMFCS level of 2, seizure disorder or a hearing deficit requiring amplification to understand commands^41^.

### RNA extraction, alignment and next generation sequencing

We extracted total RNA from whole blood according to the manufacturer’s instructions and removed ribosomal RNA and globin mRNA from 4 microg of total RNA. RNA extracts were sequenced using Illumina HiSeq2500 to generate 30 M, 2 × 100 bp reads/sample (Medgenome Labs Ltd, Bangalore, India). Based on the quality report of fastq files, high quality sequence (Q ≥ 30) was retained for further analysis and the low-quality sequence reads were excluded from the analysis. Adapter trimming was performed using fastq-mcf (version—1.04.676) and cutadapt (version 1.8dev). Contamination removal was performed using Bowtie2 (version—2.2.4) and the paired end reads were aligned to the reference human genome (GRCh37/hg19) using “STAR-v2.4.1d”. Coverage analysis for genes was performed with “HTSeq-v0.6.1” (reference gtf: GRCh37 version 75) and “bedtools-v2.17.0”. The aligned reads were used for estimating expression of the genes and transcripts using the cufflinks program.

## Data analysis

Data analysis was performed using R (version 4.0.0). For differential expression analysis we used DESeq2 (v1.17.18)^42^. We corrected all the p values for multiple testing using the Benjamini–Hochberg FDR method to control for type I error. The comparison of babies with adverse and good outcomes was performed first unadjusted and then adjusted for the randomisation allocation (masked as ‘A’ and ‘B’), gender and neutrophils and total lymphocyte cell counts, estimated by using CIBERSORT (deconvolution)^11,43^. The differentially expressed genes were subjected to pathway analysis (Ingenuity Pathway Analysis) and Fisher's Exact was used to assess the significance of the association between the differentially significant genes identified and the canonical pathways. Additional details of analysis are provided in the Supplementary Methods.

## Supplementary information

Supplementary file1
