# Supplementary material for: Transcriptomic profile of adverse neurodevelopmental outcomes after neonatal encephalopathy
Source: Sci Rep. 2020 Aug 4;10:13100. doi: 10.1038/s41598-020-70131-w (PMC7403382; doi:10.1038/s41598-020-70131-w)
Supplement: Supplementary file 1 — Supplementary file1 [file 41598_2020_70131_MOESM1_ESM.pdf]

**Supplemental Material**

# Transcriptomic profile of adverse neurodevelopmental outcomes after neonatal encephalopathy

Paolo Montaldo[1,2], Aubrey Cunnington[3], Vania Oliveira[1], Ravi Swamy[1], Prathik Bandya[4], Stuti Pant[1], Peter J Lally[1], Phoebe Ivain[1], Josephine Mendoza[1], Gaurav Atreja[1], Vadakepat Padmesh[5], Mythili Baburaj[5], Monica Sebastian[6], Indiramma Yasashwi[4], Chinnathambi Kamalarathnam[5], Rema Chandramohan[5], Sundaram Mangalabharathi[5], Kumutha Kumaraswami[5], Shobha Kumar[5], Naveen Benakappa[4], Swati Manerkar[7], Jayashree Mondhkar[7], Vinayagam Prakash[5], Mohammed Sajjid[5], Arasar Seeralar[6], Ismat Jahan[8], Sadeka Choudhury Moni[8], Mohammod Shahidullah[8], Radhika Sujatha[9], Manigandan Chandrasekaran[1], Siddarth Ramji[10], Seetha Shankaran[11], Myrsini Kaforou[3*], Jethro Herberg[3*], and Sudhin Thayyil[1*]

[1]Centre for Perinatal Neuroscience, Imperial College London, London, UK.
[2] Neonatal Unit, Università degli Studi della Campania "Luigi Vanvitelli", Naples, Italy.
[3]Paediatric Infectious Diseases, Department of Infectious Diseases, Imperial College London, London, United Kingdom.
[4]Neonatal Medicine, Indira Gandhi Institute of Child Health, Bangalore, Karnataka, India.
[5]Neonatal Medicine, Institute of Obstetrics and Gynaecology, Madras Medical College, Chennai, Tamil Nadu, India.
[6]Neonatal Medicine, Institute of Child Health, Madras Medical College, Chennai, Tamil Nadu, India.
[7]Neonatal Medicine, Sion Medical College, Mumbai, India.
[8]Neonatal Medicine, Bangabandhu Sheikh Mujib Medical University, Dhaka, Bangladesh
[9]Neonatal Medicine, Government Medical College, Thiruvananthapuram, Kerala, India
[10]Neonatal Medicine, Maulana Azad Medical College, New Delhi, Delhi, India.
[11]Neonatal-Perinatal Medicine, Wayne State University, Detroit, Michigan, USA.

*Equal contributions

**Data Analysis**
We estimated normalisation factors and negative binomial dispersions from the raw count data. With these estimates, we adjusted negative binomial generalised linear models for each gene by conducting likelihood tests. We corrected the p values for multiple testing using the Benjamini-Hochberg false discovery rate (FDR) method to control for type I errors. We performed statistical analysis using R (version 4.0.0) and DESeq2 (v1.17.18) R-packages.

**Deconvolution Analysis**
Each of the blood cell type expresses a distinct set of genes and therefore gives a different contribution to the overall RNA expression in whole blood. Therefore, the observed differences in gene expression sometimes can reflect the differences in the cell type composition instead of a change in the gene expression profile, which is the effect of interest [44]. With the widespread use of whole blood gene expression to measure and interpret biological changes between specific conditions, the use of deconvolution is becoming increasingly important to gain a better insight into cell-subset specific information [45-47]. We therefore evaluated the gene expression difference beyond those attributable to inter-individual variation in the proportion of different blood cell types. Deconvolution analysis was performed on RNA-seq data using Cibersort software, which deconvolves mixture by using a linear support vector regression and a machine learning approach [11]. The software requires the dataset to be deconvolved and a matrix of reference gene expression signatures for each of the blood cell types. We used the default input gene expression signature matrix of LM22, with 547 genes distinguishing 11 leukocytes subtypes [43].

Deconvolution algorithms are based on well-characterised samples, which are generally from adults and that application of deconvolution to blood from newborns has to be treated with caution[48]. Therefore, we took an approach, which simplified the blood components into two broad compartments to avoid introducing artefacts.

To study the potential importance of variation in blood cell numbers in the disease mechanism, we assessed the intensity of expression per unit volume of blood by multiplying normalized expression (FPKM) of each gene by the absolute cell count to give a composite measure (FPKM per L of blood). The analysis was then adjusted for the deconvolution results by using as input gene expression of the negative binomial generalized log-linear model, values adjusted for neutrophils and lymphocyte cell fraction counts. In order to create a lymphocyte cell fraction, we combined Naïve B cells, memory B cells, Plasma cells, CD8 T cells, CD4 T cells, resting memory CD4, activated memory CD4, Helper follicular T cells, regulatory T cells, Gamma delta T cells, resting NK and activated NK cells. In order to assess the cell fractions associations and gene expression, we assessed the correlation of the proportions of each cell blood type (as estimated by CIBERSORT) vs log CPM for the top differentially expressed genes (RGS1 and SMC4) and the differentially expressed genes of the melatonin pathway (based off analysis without adjustment for cell type). Note that the always opposite correlations in different cell types, may reflect the fact that proportion of blood cells are highly interdependent.

**Pathway analysis**
We used Ingenuity Pathway Analysis software (QIAGEN) to study the biological pathways enriched in the significantly differentially expressed genes. We performed the canonical pathway analysis of the differentially expressed genes, which fulfilled the criteria of an FDR value <0.05 and an absolute log2 fold change > 0.4. The association between the

differentially expressed genes and biological pathways was determined in two ways: 1) a ratio of the number of genes from the list of differentially expressed genes that maps to the pathway divided by the total number of genes that map to the same pathway 2) Fisher's Exact to assess the probability that the association between the differentially expressed genes and the canonical pathways was explained by chance alone. The P-values obtained were corrected for multiple testing using the Benjamini-Hochberg FDR method.

**Supplemental Figure 1.** Flow-chart of the study. A and B indicate the blinded allocation to either whole-body cooling or usual care.

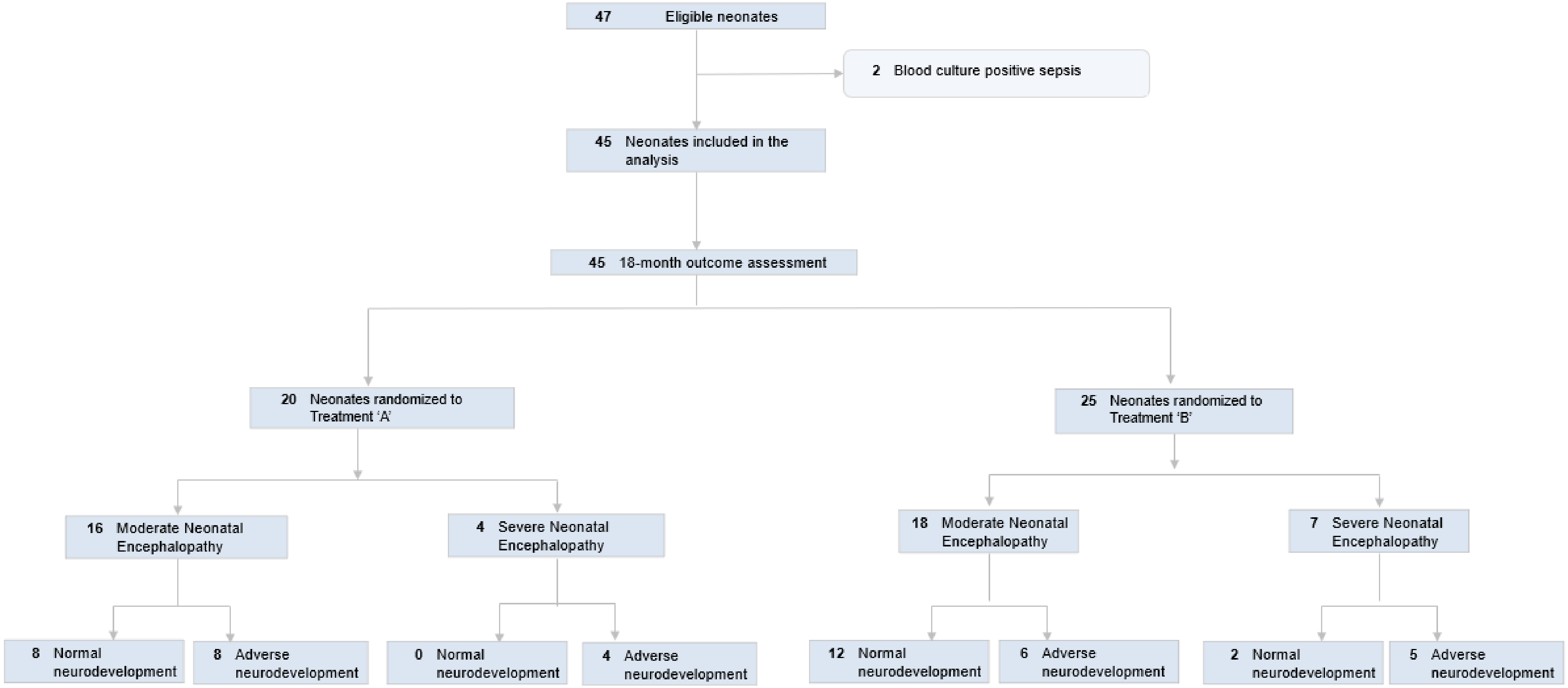

**Supplemental Figure 2.** Volcano plot showing the significant genes identified in the comparison of babies with adverse versus good outcome after adjustment for gender, trial randomization and blood cell type proportions (deconvolution), plotted according to $\log_2$ fold-change (x axis) and $\log_{10}$ p value (y axis). In green are genes with false discovery rate (FDR) < 0.05 and fold-change <0.4; in red are genes with FDR < 0.05 and fold change > 0.4.

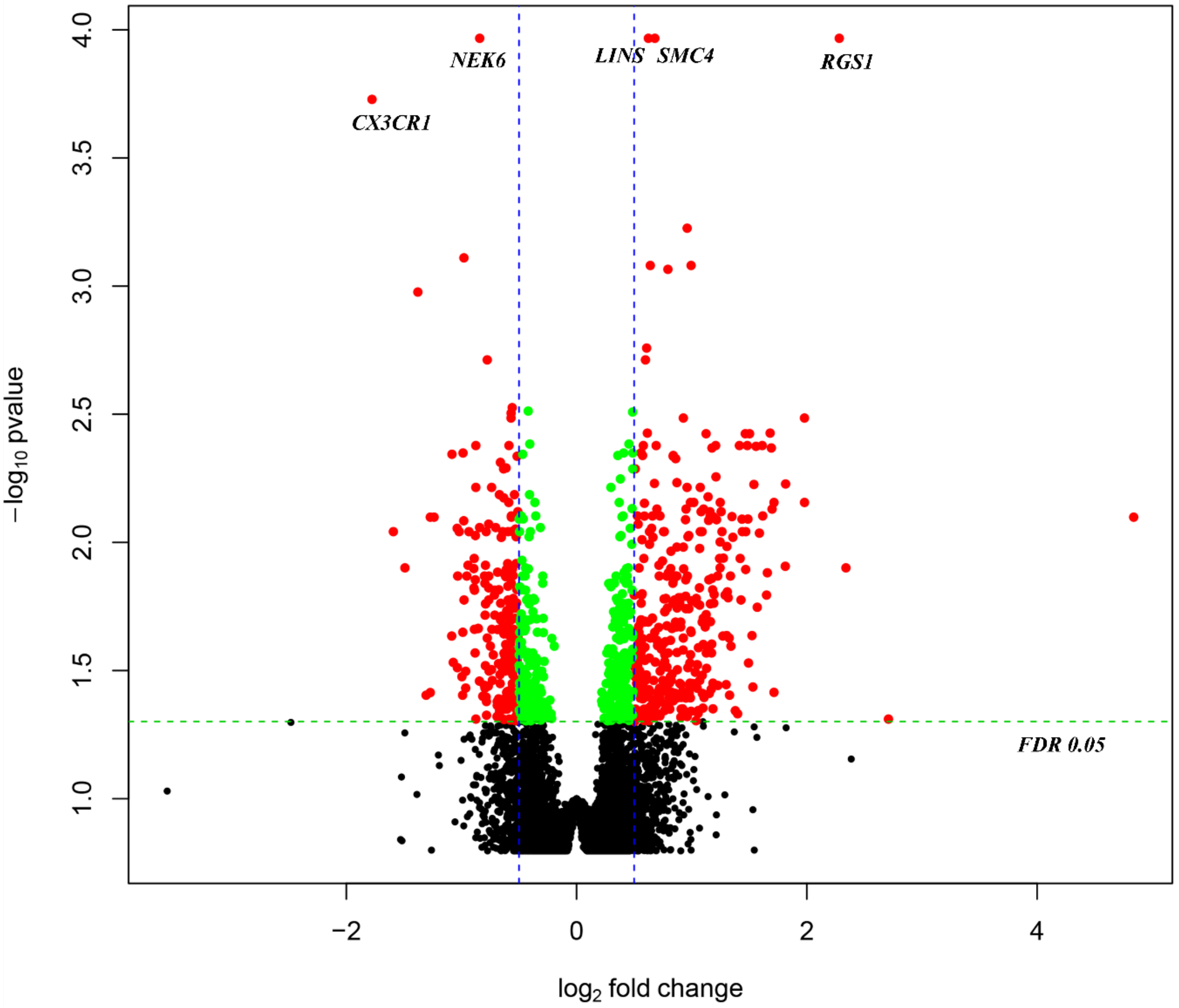

**Supplemental Table 1.** Correlation of the proportions of each cell type (as estimated by CIBERSORT) vs log counts per million for the genes of interest (based off analysis without adjustment for cell type). The relative correlation between the top differentially expressed genes and Melatonin pathway with lymphocytes and neutrophils expressed as r coefficients.

| | Lymphocytes | Neutrophils |
|---|---|---|
| RGS1 | 0.39* | -0.30* |
| SMC4 | 0.57* | -0.51* |
| CALM1 | -0.51* | 0.46* |
| CAMK4 | 0.64* | -0.48* |
| GLUT4 | 0.50* | -0.51* |
| PLCB2 | -0.04 | -0.01 |
| PLCG2 | -0.52* | 0.48* |
| PLCL1 | 0.38* | -0.28 |
| CAMK2G | -0.40* | 0.38* |
| PRKACA | -0.37* | 0.26 |
| RORA | 0.61* | -0.53* |

*p<0.05
